# Proteomics analysis of plasma protein changes in patent ductus arteriosus patients

**DOI:** 10.1186/s13052-020-00831-6

**Published:** 2020-05-19

**Authors:** Cheng Xu, Xiaoqi Su, Yong Chen, Yang Xu, Zhiqi Wang, Xuming Mo

**Affiliations:** Department of Cardiothoracic Surgery, Children’s Hospital of Nanjing Medical University, 72 Guangzhou Road, Nanjing, 210008 China

**Keywords:** Patent ductus arteriosus, Proteomics, Congenital heart disease

## Abstract

**Objective:**

Patent ductus arteriosus (PDA) is a congenital heart defect with an unclear etiology that occurs commonly among newborns. Adequately understanding the molecular pathogenesis of PDA can contribute to improved treatment and prevention. Plasma proteins may provide evidence to explore the molecular mechanisms of abnormal cardiac development.

**Methods:**

Isobaric tags for relative and absolute quantitation (iTRAQ) proteomics technology was used to measure different plasma proteins in PDA patients (*n* = 4) and controls (*n* = 4). The candidate protein was validated by ELISA and Western blot (WB) assays in a larger sample. Validation of the location and expression of this protein was performed in mouse heart sections.

**Results:**

There were three downregulated proteins and eight upregulated proteins identified in the iTRAQ proteomics data. Among these, protein disulfide-isomerase A6 (PDIA6) was further analyzed for validation. The plasma PDIA6 concentrations (3.2 ± 0.7 ng/ml) in PDA patients were significantly lower than those in normal controls (5.8 ± 1.2 ng/ml). In addition, a WB assay also supported these results. PDIA6 was widely expressed in mouse heart outflow tract on embryonic day 14.5.

**Conclusion:**

Plasma proteomics profiles suggested novel candidate molecular markers for PDA. The findings may allow development of a new strategy to investigate the mechanism and etiology of PDA.

## Introduction

Patent ductus arteriosus (PDA) is a condition in which the ductus arteriosus fails to close after birth; PDA is the second most common congenital heart disease (CHD) [1]. The incidence of PDA has been estimated to be as high as 1 in 200 births [2]. The fetal patency of the ductus arteriosus is controlled by a variety of factors, the most crucial factors are low fetal oxygen tension and cyclooxygenase-mediated metabolites of arachidonic acid (mainly prostaglandin E2 [PGE2] and prostacyclin [PGI2]) [3, 4]. Circulating PGE2 and PGI2 in the fetus cause ductus arteriosus vasodilatation by interacting with the ductal prostaglandin receptor [5]. After birth, the sudden increase in oxygen tension inhibits the voltage-dependent potassium channel in the cardiac smooth muscle, which leads to an influx of calcium and ductal contraction [6]. PGE2 and PGI2 levels decline due to metabolic lung function and elimination of their placental origin. The medial smooth muscle fibers in the heart lead to thickening of the wall, obliteration of the lumen, and shortening of the ductus arteriosus. Functional closure usually occurs within 24 to 48 h of term birth. Over the next 2 to 3 weeks, intimal folding, destruction and proliferation lead to fibrosis and a permanent seal [7]. The resulting fibrous band has no lumen and is an arterial ligament. Genetic and environmental factors may be involved in the pathogenesis of PDA [8, 9]. However, the factors underlying continuous patency of the ductus arteriosus are not fully understood.

Proteomics may play an increasingly important role in identifying biomarkers and novel pathogenic factors. Proteomics analysis of body fluids (e.g., serum and urine) is a promising tool to better understand the etiology of vascular abnormities and the pathogenesis of CHD and to explore disease markers [10–12]. Several studies have been conducted using two-dimensional electrophoresis and mass spectrometry methods to identify plasma-based biomarkers of CHD subtypes (e.g., ventricular septal defect (VSD), tetralogy of Fallot (TOF)) [13, 14]. Moreover, because proteins directly execute physiological functions, proteomics technology may be able to identify novel pathogenic factors better than genomics and metabonomics [15, 16]. However, plasma protein biomarkers for PDA, which is the second most common form of CHD, remain unknown.

Recently, isobaric tags for relative and absolute quantitation (iTRAQ) has been applied in many research fields [17, 18]. The main advantage of iTRAQ is that it can label peptides whose sites may otherwise not be accessible to other reagents at the protein level [19]. In the present study, liquid chromatography-tandem mass spectrometry (LC-MS-MS) coupled with iTRAQ was performed to quantify plasma proteins from patients with PDA and healthy controls in order to explore differential protein expression and its relationship to the pathogenesis of PDA.

## Materials and methods

### Study population and sample collection

We collected blood samples from control individuals and PDA patients preoperatively after obtaining approval from the Ethics Committee of the Children’s Hospital of Nanjing Medical University in Nanjing, China (No. 201801146–1). The control subjects were age- and gender-matched, and all subjects were the same ethnicity to reduce bias. A total of 100 controls and 100 PDA patients were included in our study. The diagnoses of all patients in the present study were confirmed by preoperative echocardiography and corrective surgery. We excluded patients with complex CHDs and complications. The legal guardian of each participant provided informed consent. The 5 mL whole blood sample of each control or patient was centrifuged at 3000 g, 4 °C for 10 min. The supernatant serum samples were stored immediately at − 80 °C for subsequent detection.

### Protein extraction, digestion and labeling

Before detection, we depleted the most abundant proteins in all eight serum samples (4 vs 4) by using the ProteoMiner protein enrichment kit (Bio-Rad Laboratories, Inc.). A total of 100 μg of protein from each sample that was 5 times volume diluted with triethylammonium bicarbonate was used for further tryptic digestion. The digestion process was performed by adding trypsin (Promega, Madison, WI, USA) to the samples with an enzyme-protein ratio of 1:50 (w/w), the mixture was then stored at 37 °C overnight. One volume of 0.1% formic acid (FA) solution (enzymatic hydrolysate) was added to acidify the proteins to peptides. Then, the peptides were desalted with a Strata-X C18 column. According to the manufacturer’s protocol, iTRAQ 8-plex kits (AB Sciex Inc., Framingham, MA, USA) were used to label the peptides. Then, a high-performance liquid chromatography (HPLC) system (Thermo DINOEX Ultimate 3000 BioRS) with a Durashell C18 (5 μm, 100 Å, 4.6 × 250 mm) was used to fractionate the labeled samples.

### Liquid chromatography-electrospray ionization-tandem mass spectrometry LC-ESI-MS/MS analysis

A TripleTOF 5600+ mass spectrometer and an Eksigent nanoLC System (SCIEX, USA) were used to dissolve each fraction with 2% acetonitrile/0.1% FA. Survey scans were acquired at 250 ms, and 30 product ion scans were gathered at 100 ms/per scan for information-dependent acquisition (IDA). The range from 350 to 1500 m/z indicated MS1 spectra, and the range from 100 to 1500 m/z indicated MS2 spectra. We excluded precursor ions for 15 s reselection.

### Enzyme-linked immunosorbent assay (ELISA)

We used an ELISA method to measure protein disulfide-isomerase A6 (PDIA6) levels. A commercial ELISA kit was purchased from MyBioSource (San Diego, USA). The lowest detection limit of the assay was 0.1 ng/ml with a coefficient of variation for the quality control specimens of < 10%.

### Western blot (WB)

Sodium dodecyl sulfate–polyacrylamide gel electrophoresis (SDS-PAGE) was conducted to separate the total proteins from the subjects’ serum samples; subsequently, the proteins were transferred to polyvinylidene fluoride membranes (Millipore, Billerica, MA, USA). The following antibody was used for the Western blot (WB) assay: anti-PDIA6 antibody (Abcam, 1:1000). Enhanced chemiluminescence (Millipore, Billerica, MA, USA) was used to test for immune complexes. Anti-albumin (Cell Signaling Technology, 1:1000) was used as an internal control. The band was semiquantified using Image Lab software (Bio-Rad laboratories, Hercules, CA, USA). Each assay was performed at least three times.

### Mice

All animal experiments were performed with the approval of the Institutional Animal Care and Use Committee of Nanjing Medical University. Six C57BL6 mice (from Shanghai SLAC Laboratory Animal Co., Ltd. Shanghai, China) were fed ad libitum diet and water and housed with a 12-h light/dark cycle at 22 °C. The mice were sacrificed at embryonic day 14.5 (E14.5).

### Immunofluorescence (IF)

Frozen sections of cardiac tissue were processed for immunofluorescence (IF) with the primary antibody to PDIA6 (1:500 dilution). Then, fluorescein isothiocyanate (FITC) dye was used to stain for PDIA6, and 4′,6-diamidino-2-phenylindole (DAPI) was used to stain nuclei. Cardiac sections were evaluated with a confocal laser scanning microscope (CLSM SP2; Leica, Nidau, Switzerland).

### Data analysis

ProteinPilot 4.5 Software (July 2012; AB Sciex) was used to identify and quantify protein levels. We searched for mass spectrometry spectra in the UniProtKB/Swiss-Prot *Homo sapiens* protein database (20,240 proteins, updated in May 2018). The parameters for the search strategy were set as follows: the instrument was TripleTOF 5600 with iTRAQ quantification and cysteine modified with iodoacetamide; the biological modifications included ID focus, trypsin digestion, and quantification; and bias correction and background correction were checked for protein quantification and normalization. Statistical significance of differences in protein expression levels between the two groups was determined by Student’s *t*-test, and multiple comparisons were corrected for with the Benjamini and Hochberg method. To identify significant changes, the threshold was set at a two-fold change with a corrected *p* value less than 0.05. ELISA and WB data were analyzed with the Mann-Whitney U test.

## Results

### Participant characteristics

The gender distribution, mean age and weight are presented in Table 1. In the iTRAQ analysis, gender was matched, and the mean ± standard deviation of age was 6.3 ± 2.0 months in control participants and 6.8 ± 2.1 months in PDA patients. In the ELISA analysis, the gender distribution and mean age were similar between two groups. No significant differences were found between the PDA and control groups in iTRAQ proteomics and ELISA analyses.

**Table 1:** Characteristics of the study subjects

	Groups	No.	Boy	Girl	Age (month)	Weight (kg)
iTRAQ	Controls	4	2 (50%)	2 (50%)	27.3 ± 7.5	11.3 ± 1.3
	PDA	4	2 (50%)	2 (50%)	29.5 ± 9.8	11.3 ± 2.3
ELISA	Controls	96	34 (35.4%)	62 (64.6%)	24.9 ± 27.1	10.7 ± 4.3
	PDA	96	35 (36.5%)	61 (63.5%)	24.1 ± 25.6	10.4 ± 5.7

*iTRAQ* Isobaric tag for relative and absolute quantitation; *ELISA* Enzyme-linked immunosorbent assay; *PDA* Patent ductus arteriosus

N (%), mean ± standard deviation

### Proteomics analysis

**Fig. 1:**
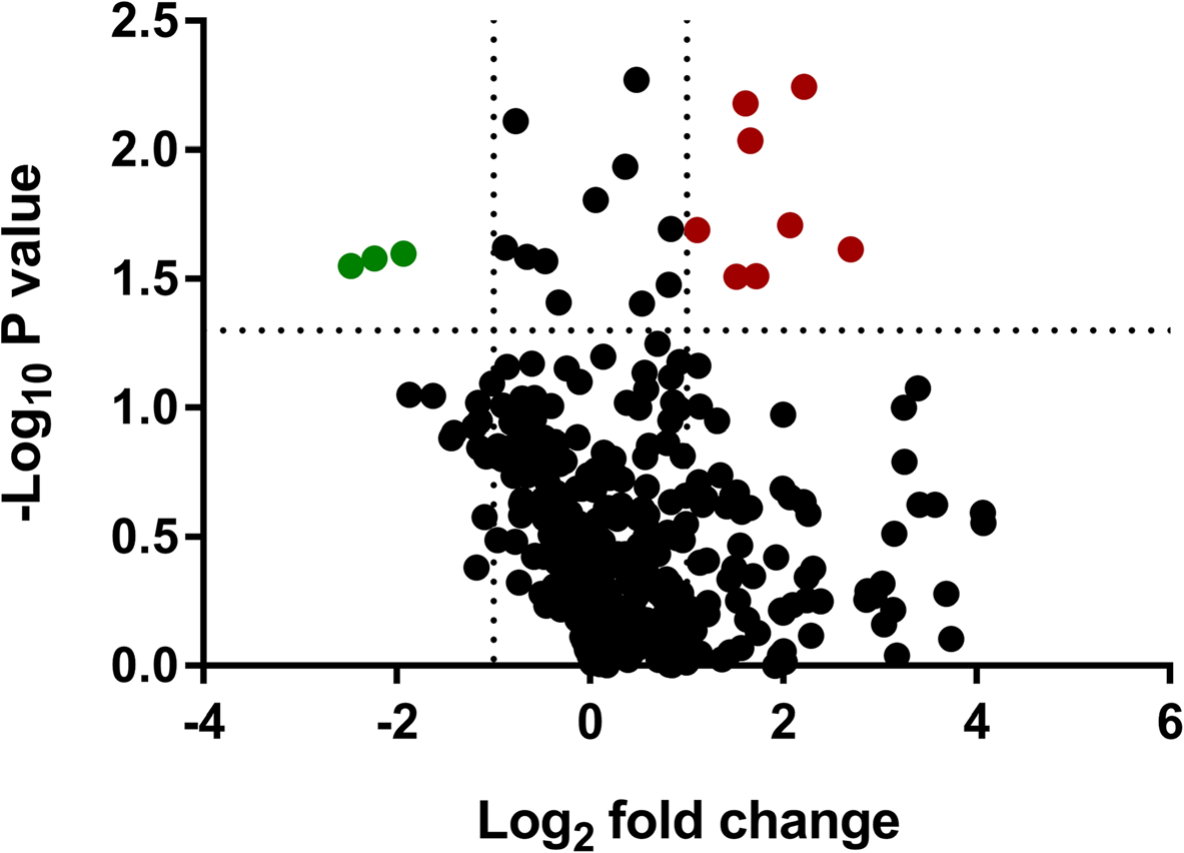
Volcano plot of proteins detected by iTRAQ among PDA patients and controls. The green dot represents decreased protein levels (*p* < 0.05 and fold-change< 0.5), and the red dot represents increased protein levels (*p* < 0.05 and fold-change> 2.0)

In this part of study, 4 plasma samples from healthy control participants and 4 plasma samples from PDA patients were collected and compared. Three proteins were significantly downregulation (*p* < 0.05 and < 0.5-fold change), and eight proteins were significantly upregulated (*p* < 0.05 and > 2.0-fold change) (Fig. 1). The details of the different protein expression levels are listed in Table 2.

**Table 2:** Differential proteins expressed in two groups with PDA compared to the control

Accession	Abbreviation	Protein Name	PDA vs Control Fold change	*P* value
P01764	HV323	Immunoglobulin heavy variable 3–23	4.64	0.005685
P12259	FA5	Coagulation factor V	3.05	0.006601
A2NJV5	KV229	Immunoglobulin kappa variable 2–29	3.15	0.009199
P01699	LV144	Immunoglobulin lambda variable 1–44	4.19	0.01957
P67936	TPM4	Tropomyosin alpha-4 chain	2.16	0.020354
Q08380	LG3BP	Galectin-3-binding protein	6.49	0.024261
P01833	PIGR	Polymeric immunoglobulin receptor	3.29	0.030769
P01700	LV147	Immunoglobulin lambda variable 1–47	2.86	0.030875
P02765	FETUA	Alpha-2-HS-glycoprotein	0.26	0.025121
Q15084	PDIA6	Protein disulfide-isomerase A6	0.21	0.02597
P00736	C1R	Complement C1r subcomponent	0.18	0.028103

### ELISA and WB

**Fig. 2:**
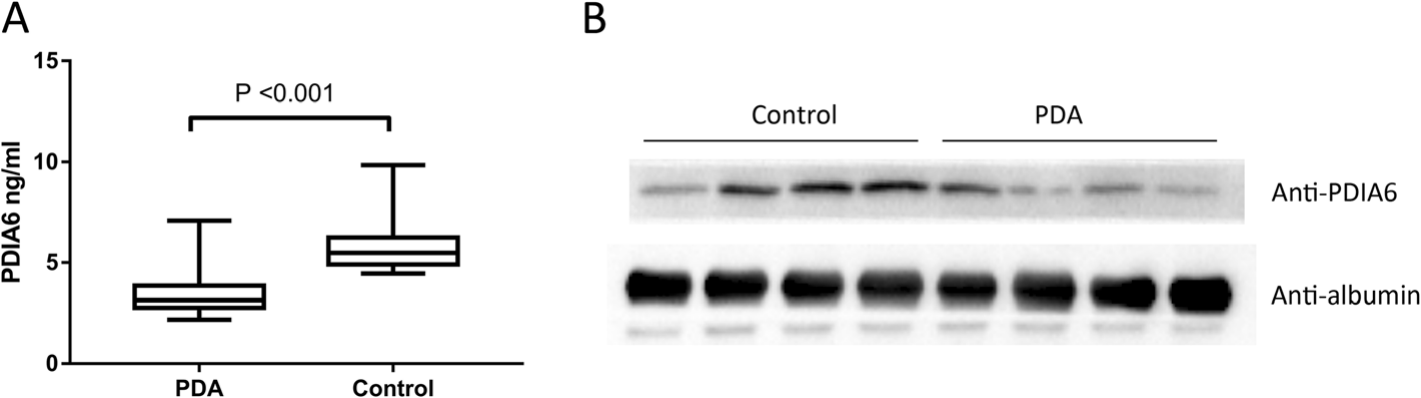
PDIA6 protein was measured by ELISA and WB in PDA patients and controls. **a**, ELISA was used to examine each subject’s plasma levels of PDIA6. *N* = 96 PDA patients and *N* = 96 controls. **b**, WB was performed to compare PDIA6 expression in plasma samples from PDA patients and controls. ELISA and WB assays were both performed three times

To validate the proteomics data, we further determined PDIA6 levels with ELISA in a larger number of samples (*n* = 96 PDA patients and *n* = 96 controls). The plasma PDIA6 concentrations in healthy controls and PDA patients were 5.8 ± 1.2 ng/ml and 3.2 ± 0.7 ng/ml, respectively (Fig. 2a). Importantly, the PDIA6 protein expression was confirmed to be significantly decreased in PDA patients compared with that in the controls (*p* = 0.004 for the Mann-Whitney U test). In addition, we also performed a WB assay to confirm the proteomics data. The plasma protein expression of PDIA6 in 4 selected PDA patients was remarkably decreased compared with that in the controls (Fig. 2b).

### Conservation comparisons of amino acids and IF

**Fig. 3:**
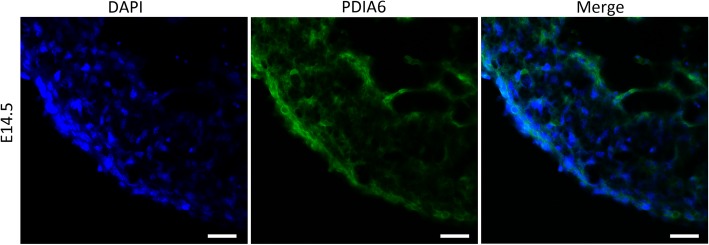
Representative IF picture showing the location and expression of PDIA6 in mouse heart tissue on E14.5. Blue indicates the nucleus, and green indicates PDIA6. The experiment was performed three times. Scar bar = 100 μm

We evaluated PDIA6 expression in mouse cardiac tissue on P0 because the samples that were previously evaluated were from circulating plasma, not tissue. First, we evaluated amino acid conservation between human, mouse and rat tissues. The amino acid sequence homology of these three species was 95% (Supplemental Figure 1). Next, we used a mouse model to represent a human model. Figure 3 shows that PDIA6 was widely expressed in the mouse hearts in E14.5.

## Discussion

In the present study, we explored changes in protein expression in plasma samples from PDA patients with iTRAQ proteomics technology. We discovered that circulating PDIA6 was downregulated in PDA patients compared with in healthy controls. PDIA6 was expressed in the ductus arteriosus of mice on P0 and may play a role in the pathogenesis of PDA.

Previously published studies on CHD (Table 3) have mainly focused on VSD and TOF subtypes of CHD. In the VSD subtype, haptoglobin, amyloid P component, carbamoyl-phosphate synthase I, and ficolin-3 have been found to be significantly decreased in patients. Haptoglobin, amyloid P component, and ficolin-3 have been reported to be associated with innate immune system function [20, 21], suggesting that CHD patients have attenuated immune function. Carbamoyl-phosphate synthase I plays a role in the urea cycle and in endogenous nitric oxide production [22]. Differential protein expression has not been reported in the PDA subtype of CHD before. We found that ELISA methods were used to confirm high-throughput results in most previous articles. We also performed WB experiments in the present study. Disparate differential protein expression results were discovered in our study and in other studies. We speculate that different populations, proteomics technology, and disease subtypes evaluated may be the reason for these differences. The present research revealed an association between differential protein expression and PDA; however, in vitro and in vivo models may provide more evidence to demonstrate a causal relationship.

**Table 3:** Published proteomics study in congenital heart disease (CHD)

Sample source	Subtype of CHD	Proteomics technology	Confirm method	Compare	Up	Down	References (PMID)
Children plasma	VSD	2DE	ELISA	VSD vs. control	Orosomucoid 2	Haptoglobin, amyloid P-component	25,914,298
Children plasma	VSD, ASD	iTRAQ	ELISA	VSD vs. control, ASD vs. control	N/A	Carbamoyl-phosphate synthase I, Complement Factor H-related Protein 2	27,886,187
Children plasma	TOF	2DE	ELISA	TOF vs. control	N/A	Gelsolin, Ficolin-3	24,565,402
Children plasma	VSD	2DE	ELISA	VSD vs. control	N/A	Ficolin-3	24,565,402
Maternal serum	TOF	LC/MS	N/A	TOF vs. control	N/A	N/A	28,598,000

*TOF* Tetralogy of Fallot; *VSD* Ventricular septal defect; *ASD* Atrial septal defect; *2DE* Two-dimensional electrophoresis; *LC/MS* Liquid chromatography/mass spectrometry; *ELISA* Enzyme-Linked Immunosorbent Assay; *N/A* Not applicable

PDIA6 is located in the eukaryotic ER and functions as an isomerase and molecular chaperone [23]. Several reports using loss-of-function assays have shown that PDIA6 plays a role in unfolded protein response signaling [24] and acts as a negative modulator of both Ser/Thr protein kinase inositol-requiring enzyme-1 and protein kinase RNA-like ER kinase [25]. In addition, PDIA6 maintains calcium homeostasis in the ER [26]. The ER can affect cardiac development and function in the following ways: a) acting in Ca^2+^-dependent pathways; b) playing a role in folding proteins; c) targeting membrane-bound and secretory proteins; and d) responding to cellular stress events, such as hypoxic conditions [27]. Several ER genes are activated during the early stages of cardiogenesis, for example calreticulin-1 [28], Grp94 [29], and BiP [30], suggesting the potential function of the ER in embryonic cardiac development. Vekich et al. found that PDIA6 could protect cardiomyocytes by interacting with activating transcription factor 6 [31]. However, the exact roles of PDIA6 in cardiac development remain unclear.

There are several limitations in our study. First, although the number of PDA patients included was large, a multicenter study may yield more convincing results. Additionally, examination indicators such as echocardiography were not obtained in this study, and the associations between altered protein expression and clinical parameters may reveal underlying pathogenic mechanisms. In addition, long- term follow-up data may further elucidate the roles of PDIA6. Finally, because the study was a cross-sectional study, it was not possible to determine whether PDA led to reduced plasma PDIA6 concentrations or whether decreased PDIA6 concentrations induced PDA progression. Furthermore, we will use a loss-of-function method in vitro and in vivo to explore whether PDIA6 plays a role in the pathogenesis and molecular mechanisms of PDA.

## Conclusion

In conclusion, we identified, for the first time, found alterations of 11 differential proteins by using the iTRAQ in the plasma of patients with PDA and controls. Importantly, our result demonstrated that the level of plasma PDIA6 was downregulated in patients with PDA, which may partially explain the roles of PDIA6 in cardiac development. The altered PDIA6 we identified may have potential clinical implications for PDA treatment and provide evidence regarding the etiology and molecular mechanism of PDA.

## Supplementary information

**Additional file 1.**

## Data Availability

All data analysed during this study are included in this published article and its supplementary information files.
