# Supplementary figures and images for: Proteomics analysis of plasma protein changes in patent ductus arteriosus patients
Source: Ital J Pediatr. 2020 May 19;46:64. doi: 10.1186/s13052-020-00831-6 (PMC7236322; doi:10.1186/s13052-020-00831-6)

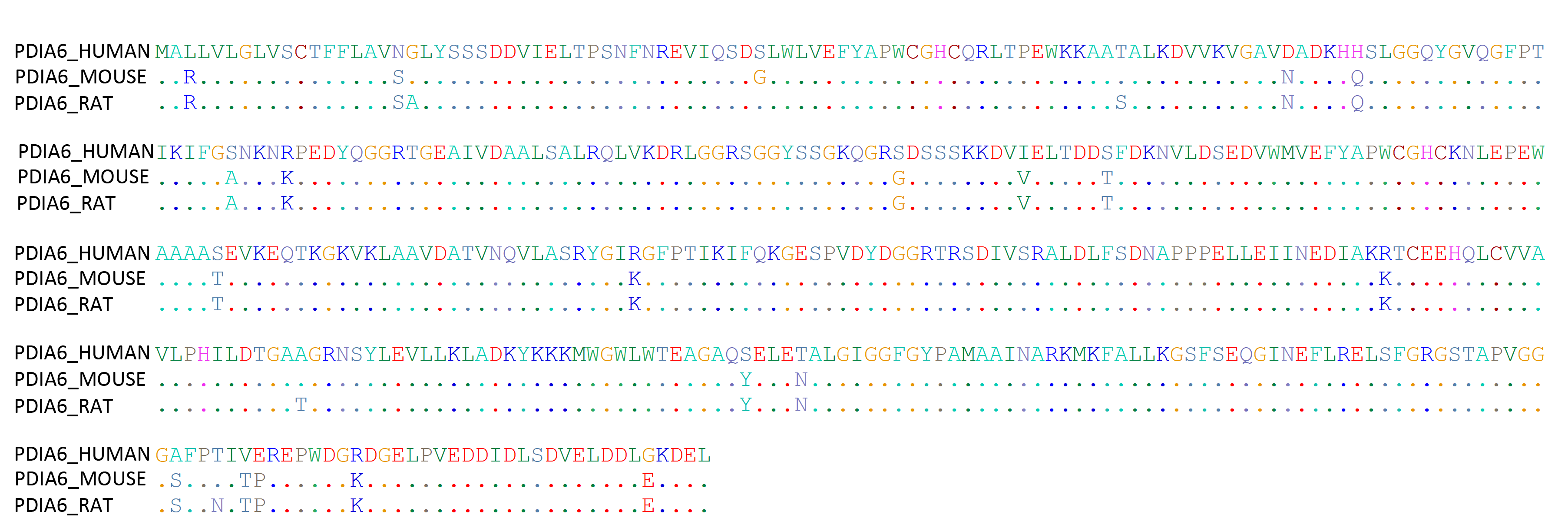

Supplement: Supplementary file 1 — Additional file 1. [file 13052_2020_831_MOESM1_ESM.tif]
